# The Burden of Anemia in Pregnancy Among Women Attending the Antenatal Clinics in Mkuranga District, Tanzania

**DOI:** 10.3389/fpubh.2021.724562

**Published:** 2021-12-02

**Authors:** Evelyine B. Ngimbudzi, Siriel N. Massawe, Bruno F. Sunguya

**Affiliations:** ^1^School of Public Health and Social Sciences, Muhimbili University of Health and Allied Sciences, Dar es Salaam, Tanzania; ^2^School of Medicine (S.N.M.), Muhimbili University of Health and Allied Sciences, Dar es Salaam, Tanzania

**Keywords:** antenatal (ANC), nutrition, anemia, dietary diversity, food security

## Abstract

**Introduction:** The burden of anemia in pregnancy is of global health importance. Tanzania is no exception. Its effects vary from one region to another due to the differing causes. Overall, it is a significant cause of maternal mortality. This study sought to assess the prevalence and factors associated with anemia among pregnant women attending the antenatal clinic (ANC) in the Mkuranga district of the Pwani region of Tanzania.

**Methodology:** This cross sectional study was conducted among 418 pregnant women aged 15–49 years attending the Mkuranga District Hospital and Kilimahewa Health Center. The outcome variable of interest was anemia in pregnancy defined as a hemoglobin concentration of 11 g/dl or less. Data was collected using face-to-face interviews with a standardized pretested questionnaire, and through blood samples collected for hemoglobin testing. Descriptive analysis was used to determine the prevalence of anemia while multiple logistic regression was used to determine factors associated with anemia in pregnancy.

**Results:** Anemia was prevalent among 83.5% of pregnant women attending the two major ANCs in Mkuranga district. Categorically, the hemoglobin of 16.3% of the included women was normal, 51.9% had moderate anemia, 24.4% had mild anemia, and 7.2% had severe anemia. Factors associated with anemia included being in the third trimester (AOR = 2.87, *p* = 0.026), not consuming vegetables (AOR = 2.62, *p* = 0.008), meat (AOR = 2.71, *p* = 0.003), eggs (AOR = 2.98, *p* = 0.002), and fish (AOR = 2.38, *p* = 0.005). The finding of unadjusted analysis revealed that women with inadequate minimum dietary diversity were having significantly greater odds of being anemic as compared with those with adequate dietary diversity (OR = 1.94, *P* = 0.016).

**Conclusion:** More than 80% of pregnant women attending ANC in Mkuranga districts were anemic. Such unprecedented burden of anemia is associated with several factors, which include poor dietary practices such as not consuming iron-rich foods, for example vegetables, meat, eggs, and fish. Women in their third trimester were also more likely to suffer from anemia. This unprecedented burden of anemia in pregnancy can be addressed if efforts to improve feeding practices and early monitoring at the ANCs are sustained.

## Introduction

About one in four women conceive with inadequate or absent iron stores with the levels of serum ferritin below 30 mg/l, and up to 90% have iron stores of below 500 mg, or with serum ferritin below 70 mg/l (1). These levels are insufficient to meet the increased iron needs during pregnancy, delivery, and postpartum. Moderate to severe anemia in pregnancy especially at 28 weeks and above contributes to 23% maternal mortality globally (2). It is associated with parasitic diseases such as malaria and worm infestations, acute or chronic illnesses such as sickle cell anemia, tuberculosis, HIV infection, and different macronutrient disorders (3–6).

Anemia is prevalent among 57.1% of pregnancies in Africa (7). It is more common in Sub-Saharan Africa owing to a lower intake of iron and other micronutrients before and during pregnancy (8). Pregnancy is an iron-demanding period due to a growing fetus and changing physiological status. The deficiency of iron during this period remains one of the risk factors for maternal mortality and overall mortality in the general population (6).

Evidence suggests that 45% of all women of reproductive age in Tanzania are anemic (9). The incidence varies between and within regions. It ranges from 25% in the Mbeya region to 72% in the Kaskazini Pemba. Moreover, the burden is higher among pregnant women (57.1%) compared with the general population (9). Routine administrative hospital records and surveys from Mkuranga district hospital suggest that anemia in pregnancy is a leading contributory factor among cases that are admitted to the maternity ward. In absolute numbers, the total number of admissions in the maternity ward were 4,800 and among of them 3,087 were admitted due to anemia in 2017.

Anemia in pregnancy has several maternal health effects such as preterm deliveries, heart failure, postpartum hemorrhage, and even death (4). For the fetuses, the effects include low birth weight, birth asphyxia, and perinatal death (10–12). Babies born from anemic mothers are at a greater risk of being impaired mentally, physically, and exhibit poor school performance later (13). Also, preterm infants are likely to have growth retardation and evidence of low iron stores in their first year of life (14). Anemia in pregnancy can therefore pose long-term consequences in the national economic development through low education attainment, reduced quality of life, decreased level of economic productivity, and therefore a cycle of poverty (15).

Ensuring quality health care services in antenatal clinics (ANCs) can help in addressing anemia and other pregnancy-related challenges (16). ANCs are designed to provide an opportunity to pregnant women for a variety of health care services including health education, counseling, screening, treatment, monitoring, and promoting the well-being of the mother and the fetus (16). Many strategies have been implemented in the country to ensure pregnant women are receiving quality antenatal services concerning the prevention, diagnosis, and treatment of anemia. Such strategies include testing of hemoglobin level in every antenatal visit, intermittent preventive treatment in pregnancy (IPTp) for malaria, provision of insecticide-treated bed nets (ITN), ferrous sulfate tablet, and deworming. These services are targeted to be provided to every pregnant woman within the country through ANCs.

The World Health Assembly set six targets to be accomplished by the year 2025. Among the targets is a 50% reduction of anemia in women of reproductive age through several strategies such as food fortification with iron, folic acid, and other micronutrients, distribution of iron-containing supplements, control of infections and malaria (15). Previous studies suggest that associated factors for anemia in pregnancy vary between and within regions. Since anemia is reported to be number one among all cases that are admitted in Mkuranga district hospital maternity ward, and because no study has been conducted to address the problem there is a need to identify the magnitude and factors associated with it.

## Methodology

### Study Setting

The study was conducted among women attending ANCs at Mkuranga District Hospital and Irene-Kilimahewa Health Center coastal region in Tanzania. The two facilities were selected based on their location and the number of villages that they serve. Mkuranga District Hospital is located at Mkuranga Center (town) whereas Irene Kilimahewa is located 36 km from Mkuranga Center. Mkuranga District Hospital RCH provides services to seven villages within the districts and also it is a referral center for all health centers in the district, whereas Irene-Kilimahewa serves eight villages and it receives referrals from 10 dispensaries within the district. Around 257 (61.4%) of the participants were from Mkuranga District Hospital and 161 (38.5%) of the participants were from Kilimahewa Health Center. The proportion of numbers were based on the total number of clients attending ANC per day. The two facilities are homogeneous in nature since they receive people of the same culture, and they all provide blood transfusion services. Therefore, the two facilities were selected based on the factors explained above.

### Study Design and Sampling Method

The study population was pregnant women of reproductive age (that is women ages 15–49 years). The sample size was estimated by using Fisher's formula (17) *n* = Z^2^ P (1-P)/ε^2^, where *n* is the estimated minimum sample size; *Z* is the confidence level at 95% (standard value is 1.96); *P* is the proportion (prevalence of anemia during pregnancy 53% TDHS, 2010); and ε is the precision at 95% CI = 0.05. The minimum sample that was required for this study was 399 pregnant women. A 5% non-response rate was used to give a total sample size of 418 pregnant women. The study had no dropouts, and hence all the 418 pregnant women were included.

### Inclusion and Exclusion Criteria

The study included pregnant women attending ANC from the first visit onwards at the Irene Kilimahewa Health Center and Mkuranga District Hospital RCH-ANC. The study excluded pregnant women who did not start their first visit at Irene Kilimahewa Health Center and Mkuranga District Hospital (relocate). In addition, pregnant women who were not able to express themselves in either Kiswahili or English were excluded as participants in the study.

### Data Collection

Pregnant women who were aged between 15 and 49 years attending ANC at Mkuranga District Hospital and Irene Kilimahewa Health Center RCH-ANC were included. The study involved 418 participants who were conveniently sampled. Data were collected through structured questionnaires and blood sampling. The purpose of the study was explained to all eligible individuals. Those who agreed to participate were asked to sign the consent form.

#### Tools and Questionnaires

A questionnaire with structured questions was used to collect data that assessed factors associated with anemia in pregnancy. The outcome variable of the study was anemia in pregnancy where all pregnant women who were found with a hemoglobin level <11 g/dl were considered to be anemic (6). According to WHO, anemia in pregnancy is categorized into three groups whereby those with a hemoglobin level of 10.0–10.9 g/dl are considered to have mild anemia, 7.0–9.9 g/dl moderate anemia, and <7 g/dl severe anemia (18). Independent variables of the study were socio-economic and sociodemographics variables that were assessed by 34 questions adopted from Tanzania demographic health survey (9).

Household food insecurity was assessed by using the tools adopted from FANTA and WHO (19). The tool has nine questions that required the woman to recall her eating experience in the previous month. The average Cronbach's alpha reliability coefficient for the instrument was 0.76. The lowest and highest values were 0 and 27, respectively. The scores were grouped into four categories: food secure, mildly insecure, moderately insecure, and severe food insecurity as recommended by the developer of the questionnaire based on cut-off points. The tool was validated in developing countries including Tanzania (20).

#### Dietary Diversity

Pregnant women were asked to identify the type of food they took in the previous 24 h. A list of common foods was adopted from the tool developers. A list of 10 food groups provided by FANTA (21) was used to calculate the dietary diversity score (WDDS) of women. Minimum dietary diversity was defined as it was instructed by the tool developers. The women who consumed five meals and above were considered to have minimal adequate dietary diversity. Also, the tool was validated (22).

#### The Burden of Disease

The impact of anemia in relation to other health conditions, such as malaria, was assessed through questions that were adopted from TDHS/MIS 2016. The tool was validated by the previous users within the country.

### Data Analysis

The analysis was conducted using STATA version 15. All probabilities were two-tailed and independent variables with *p*-values < 0.05 were regarded as significantly related to anemia. Descriptive statistics involving cross tabulations were used to analyze categorical variables, and results were presented in the form of frequency and percentage, whereas mean and SD were presented for continuous variables. Logistic regression analysis was applied to determine factors associated with anemia among pregnant women. Bivariate regression was first fitted for each study variable to identify the independent variables that were associated with anemia. Variables that were significant in bivariate analysis with (*P* = 0.05) were then included in a multivariate analysis to obtain the adjusted factors associated with anemia. The results of the model were presented using odds ratios (OR) and 95% confidence interval (CI).

## Results

### Sociodemographic and Economic Characteristics of Study Participants

A total of 418 pregnant women aged between 15 and 49 years were included in this study (Table 1). Women had a median age of 25 years with a SD of 6.83 years. About (26.2%) of the respondents were aged 20–24 years. The majority of participants (84.3%) were married. About 76.6% of the women reported to having enrolled in formal education at least once in their lifetime. Most of the participants (54.3%) were involved in agriculture activities. Participants had a fairly equally distributed wealth category. The majority, which accounts for 225 (53.8%), were in the third trimester of their pregnancy. Out of the total participants, 257 (61.5%) were in their second trimester at first ANC. The results of malaria tests were found to be negative in 359 (86.1%) participants. Of the entire participants, the majority 173 (41.4%) had one to two children in the household. More than 90% reported having slept under the net the previous night. The majority (76.9%) of the households experienced severe food insecurity and 10.3% had food security.

**Table 1:** The background characteristics of the pregnant women under the study.

**Variable**	**Frequency (*n*)**	**Percent (%)**
Age (years)		
<19	71	16.99
20–24	122	29.19
25–29	96	22.97
30–34	69	16.51
35–39	42	10.05
40+	18	4.31
Education level		
No formal education	98	23.44
Primary education	241	57.66
Secondary or higher	79	18.90
Current marital status		
Married or living together	346	82.78
Not married	72	17.22
Financial activity		
Profession/Technical/Managerial	3	0.72
Clerical	1	0.24
Sales and services	20	4.78
Skilled Manual	20	4.78
Unskilled Manual	75	17.94
Domestic services	21	5.02
Agriculture	227	54.31
Not employed	51	12.20
Wealth index quantiles		
Lowest	86	20.57
Lower	82	19.62
Middle	83	19.86
Higher	85	20.33
Highest	82	19.62
Pregnancy trimester		
1st trimester	47	11.24
2nd trimester	146	34.93
3rd trimester	225	53.83
Pregnancy trimester at first ANC visit		
1st trimester	143	34.21
2nd trimester	257	61.48
3rd trimester	18	4.31
Malaria test results		
Positive	58	13.91
Negative	359	86.09
Number of children in household		
None	140	33.49
1–2	173	41.39
3+	105	25.12
Sleep under treated net last night		
Yes	398	95.22
No	20	4.78
Food insecurity status		
Food secure	43	10.34
Mild food insecure	12	2.88
Moderately food insecure	42	10.10
Severely food insecure	319	76.68

**Figure 1:**
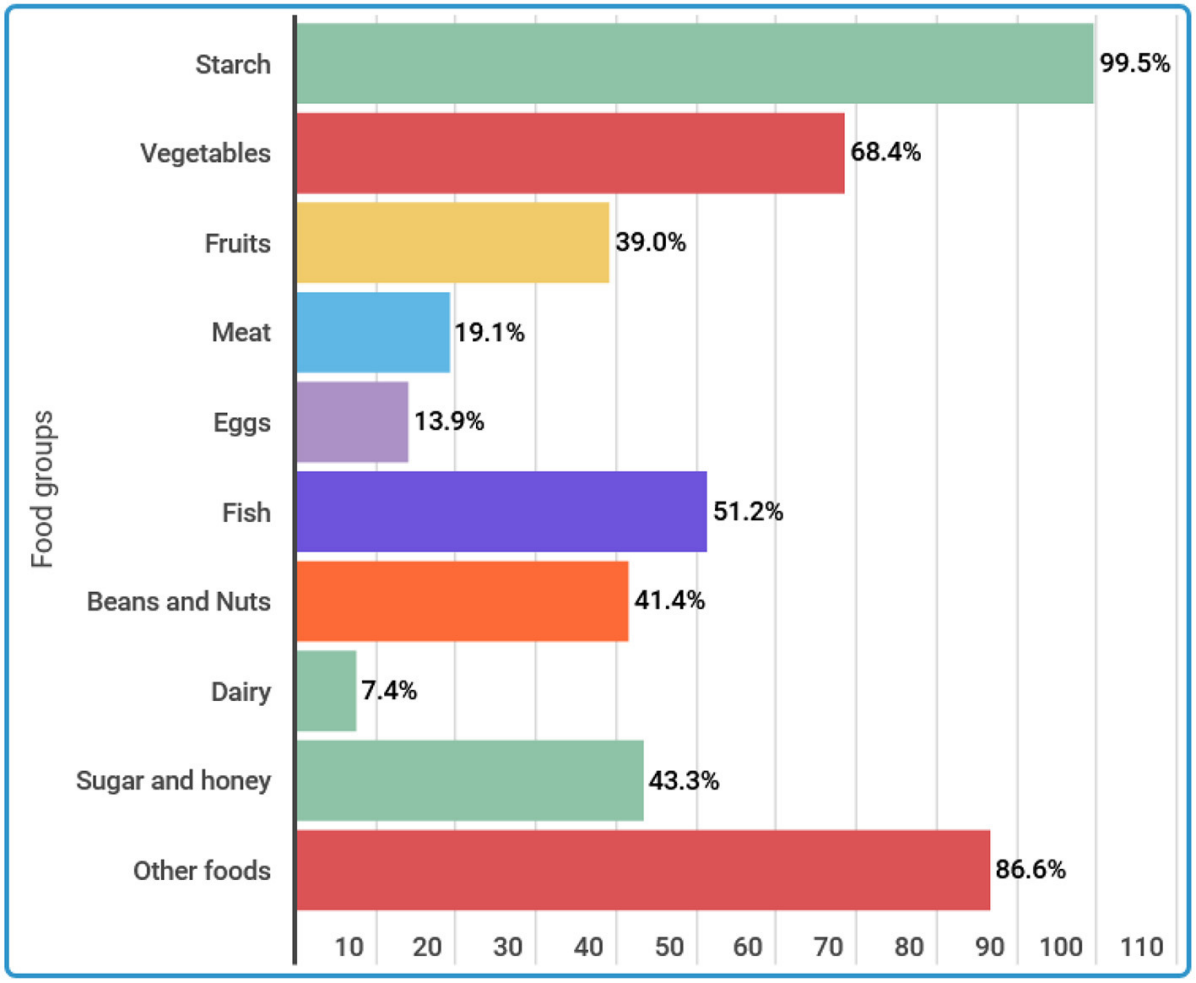
Type of food consumed by women within 24 h before the interview.

**The 24-h recall woman dietary diversity score (WDDS)** findings showed that the overall mean dietary diversity score was 4.70 with a SD of 1.41. This means that on an average, each woman consumed five different food groups within 24 h before the survey. The minimum and maximum WDDS was 1 and 9, respectively. Categorically, more than 50% (51.2%) of the participants consumed the required minimum dietary diversity for women. Figure 1 displays the seven food groups consumed by women within 24 before the survey. The most consumed food group was starch (99.5%) followed by other foods (86.6%) and vegetables food groups (68.4%). Besides, more than half (51.2%) consumed fish, 41.4% consumed beans and nuts, whereas 13.9% consumed eggs within 24 h.

### Prevalence of Anemia Among Pregnant Women

**Figure 2:**
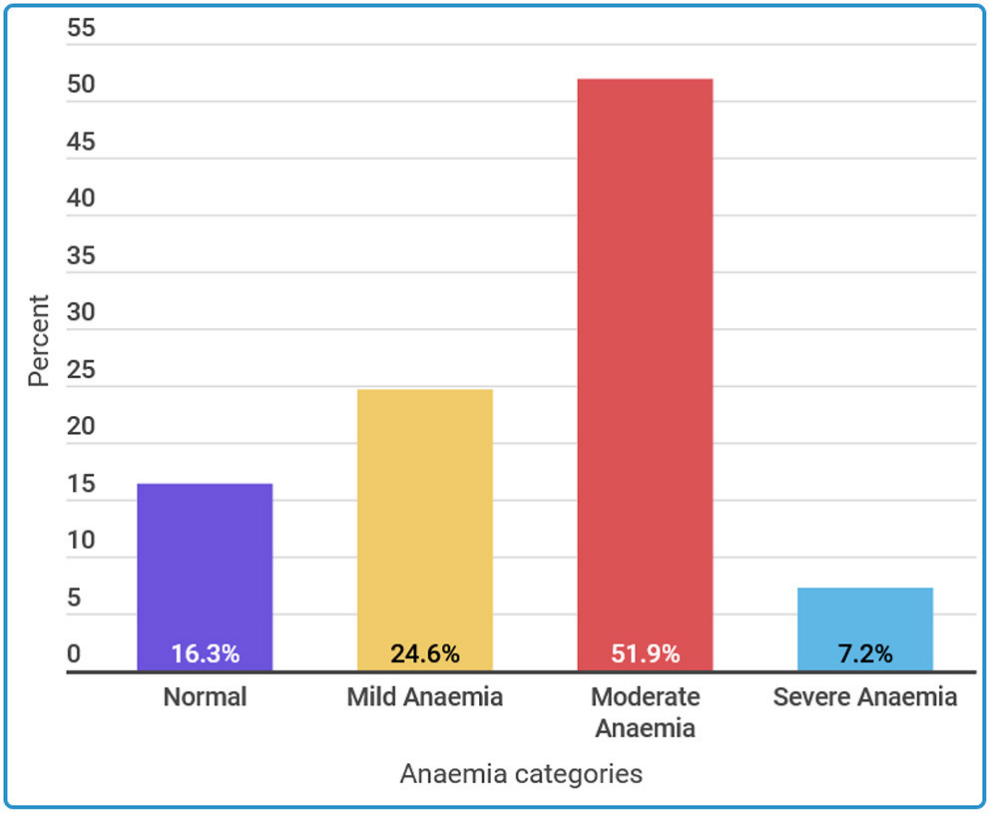
Prevalence of anemia among pregnant women.

Hemoglobin measurements were taken. The mean hemoglobin level was reported to be 9.5 g/dl with a SD of 1.6 g/dl. The overall magnitude of anemia (hemoglobin level < 11 g/dl) was 83.7%. Categorically, 16.3% were normal, 51.9% had moderate anemia, 24.4% had mild anemia, and 7.2% had severe anemia (Figure 2).

### Factors Associated With Anemia Among Pregnant Women

Social demographic and socioeconomic factors of the pregnant women were compared with the anemic status. In bivariate analysis, the associations between individual independent variables and dependent variables (anemia) did not reach a statistical difference of 5% level of significance (Table 2). The prevalence of anemia was higher among women who were currently married or living together with a man (81.4%) as compared with unmarried counterparts (18.6%), although this difference did not reach a statistically significant level. In general, women who attended secondary or higher education had a lower burden of anemia (18.0%) compared with those with no formal education (23.4%) and primary education level (58.6%). In this study, the prevalence of anemia was evenly distributed among wealthy quintiles. However, these results showed no statistically significant association in both bivariate and multivariate analyses.

**Table 2:** Bivariate and multivariate analysis of factors associated with anemia among pregnant women attending ANC in Mkuranga district hospital.

**Variable**	**Anemia**, ***n*** **(%)**		**OR (95%, CI)**	***p*-value**	**AOR (95%, CI)**	***p*-value**
	**No**	**Yes**				
**Age**				0.463		0.620
<19	8 (11.8)	63 (18.0)	Reference		Reference	
20-24	17 (25.0)	105 (30.0)	0.78 [0.32, 1.92]	0.595	0.66 [0.25, 1.76]	0.411
25-29	17 (25.0)	79 (22.6)	0.59 [0.24, 1.46]	0.252	0.77 [0.28, 2.14]	0.615
30-34	15 (22.1)	54 (15.4)	0.46 [0.18, 1.16]	0.100	0.50 [0.17, 1.41]	0.191
35-39	9 (13.2)	33 (9.4)	0.47 [0.16, 1.32]	0.150	0.37 [0.11, 1.23]	0.108
40+	2 (2.9)	16 (4.6)	1.02 [0.19, 5.26]	0.985	0.68 [0.11, 4.18]	0.682
**Education level**						
No formal education	16 (23.5)	82 (23.4)	Reference			
Primary education	36 (52.9)	205 (58.6)	1.11 [0.58, 2.11]	0.748		
Secondary or higher	16 (23.5)	63 (18.0)	0.77 [0.36, 1.65]	0.500		
**Current marital status**						
Married or living together	61 (89.7)	285 (81.4)	Reference		Reference	
Not married	7 (10.3)	65 (18.6)	1.99 [0.87, 4.55]	0.104	2.04 [0.82, 5.07]	0.124
**Wealth index quintiles**						0.724
Lowest	11 (16.2)	75 (21.4)	Reference		Reference	
Lower	11 (16.2)	71 (20.3)	0.95 [0.39, 2.32]	0.905	0.84 [0.31, 2.26]	0.737
Middle	13 (19.1)	70 (20.0)	0.79 [0.33, 1.87]	0.593	0.99 [0.37, 2.64]	0.983
Higher	19 (27.9)	66 (18.9)	0.51 [0.23, 1.14]	0.104	0.65 [0.26, 1.63]	0.362
Highest	14 (20.6)	68 (19.4)	0.71 [0.30, 1.67]	0.437	1.20 [0.44, 3.23]	0.722
**Pregnancy trimester**				**0.001**		**0.023**
1st trimester	16 (23.5)	31 (8.9)	Reference		Reference	
2nd trimester	26 (38.2)	120 (34.3)	2.38 [1.14, 4.97]	**0.021**	1.48 [0.56, 3.97]	0.430
3rd trimester	26 (38.2)	199 (56.9)	3.95 [1.91, 8.18]	**<0.001**	2.87 [1.13, 7.25]	**0.026**
**Pregnancy trimester at first ANC visit**						
1st trimester	31 (45.6)	112 (32.0)	Reference		Reference	
2nd/3rd trimester	37 (54.4)	238 (68.0)	1.78 [1.05, 3.02]	**0.032**	1.42 [0.69, 2.93]	0.342
**Malaria test results**						
Positive	0 (0.0)	58 (100.0)	370674027.1 [0.0]	0.997		
Negative	67 (18.7)	292 (81.3)	Reference			
**Household food security status**						
Food secure	8 (11.8)	35 (10.0)	1.20 [0.53, 2.71]	0.662		
Food insecure	60 (88.2)	315 (90.0)				
Dietary diversity						
Inadequate	24 (11.8)	180 (88.2)	1.94 [1.13, 3.33]	**0.016**		
Adequate	44 (20.6)	170 (79.4)	Reference			
**Consumed vegetables**						
No	12 (17.6)	120 (34.3)	2.43 [1.26, 4.72]	**0.008**	2.62 [1.28, 5.38]	**0.008**
Yes	56 (82.4)	230 (65.7)	Reference		Reference	
**Consumed meat**						
No	45 (66.2)	293 (83.7)	2.63 [1.48, 4.68]	**0.001**	2.71 [1.39, 5.25]	**0.003**
Yes	23 (33.8)	57 (16.3)	Reference		Reference	
**Consumed eggs**						
No	48 (70.6)	312 (89.1)	3.42 [1.84, 6.36]	**<0.001**	2.98 [1.47, 6.03]	**0.002**
Yes	20 (24.4)	38 (10.9)	Reference		Reference	
**Consumed fishes**						
No	21 (30.9)	183 (52.3)	2.45 [1.41, 4.27]	**0.002**	2.38 [1.29, 4.29]	**0.005**
Yes	47 (69.1)	167 (47.7)	Reference		Reference	

*OR, odd ratio; AOR, Adjusted odd ratio*.

The results of the bivariate analysis showed that pregnancy trimester at first ANC visit were significantly associated with anemia among pregnant women, *p* = 0.001 and *p* = 0.032, respectively. The association was also significant in multivariate analysis for pregnancy trimesters and trimester at the first ANC visit, *p* = 0.023 and *p* = 0.026, respectively. Women whose pregnancy was at second (OR = 2.38, *p* = 0.021) and third trimesters (OR = 3.95, *p* < 0.001) were significantly more likely to have anemia as compared to women in the first trimester. However, in multivariate analysis, only women in the third trimester were significantly associated with anemia. In addition, women who started the first ANC visit in the second or third trimester were significantly more prevalent to having anemia than women who started the ANC visit during the first trimester (OR = 1.78, *p* = 0.032). The results were however non-significant in multivariate analysis. Besides, the finding of unadjusted analysis revealed that women with inadequate minimum dietary diversity were having significantly greater odds of being anemic as compared with those with adequate dietary diversity (OR = 1.94, *P* = 0.016). Thus, the odds of being anemic among women with inadequate minimum dietary diversity was almost twice that of women with adequate minimum dietary diversity. However, the dietary diversity was not included in adjusted analysis so as to avoid multicollinearity with its individual items used to measure it. Yet the individual food groups used to create minimum dietary diversity for women that were significant in an unadjusted analysis were included in an adjusted analysis. It was also noted that women who did not consume vegetables, meat, eggs, and fish were significantly more likely to be anemic than women who consumed, OR = 2.43 *p* = 0.008, OR = 2.63 *p* = 0.001, OR = 3.42 *p* = 0.001, and OR = 2.45 *p* = 0.002, respectively. In multivariate analysis, the results were also significant.

## Discussion

We conducted a study that focused on assessing the magnitude of anemia and the factors associated with it among pregnant women attending two ANCs within the Mkuranga district. The study found that 83.7% of pregnant women attending ANC at Mkuranga district were anemic. According to WHO, classification of public health importance of anemia, the observed figure implies anemia in pregnancy is a serious public health problem in the study setting (23). The prevalence of anemia found in this study is higher compared with the national prevalence of 57.1% (9). The prevalence of this study is also higher compared to that found in Moshi (18.0%) (4) and Dar es Salaam (68.0%), Tanzania (13). These findings might be implicated with cultural practices and eating patterns of people in the coastal region since they always do not consume much vegetables and fruits in their diet, knowing or unknowing the effects that might be associated with that practice. It is reported that the prevalence of anemia in developed countries ranges between 3 and 18% whereas in developing countries it ranges between 35 and 75% (1, 4, 24).

In most studies conducted in Africa, the majority of those who were anemic were pregnant women aged 20–24 years, having a primary level of education, married, with the lowest wealth index. It was discussed in many studies that a low level of education might increase the chance of someone getting anemia due to the fact that educated women have a greater chance of getting proper information in relation to health issues like anemia. Also being educated may influence someone to comprehend the information that is provided at the ANC. The wealth status of women was also considered to be among the predicators of anemia in pregnancy, since pregnant women with low to middle wealth index were considered not able to get enough number of meals. These findings look similar to the findings from several studies conducted in Ethiopia, Ghana, and Malaysia which also indicate that pregnant women wealth status and occupation may contribute to anemia (1, 25, 26).

The majority of pregnant women who were included in this study experienced moderate anemia followed by mild anemia. These findings are similar to those from Moshi, Tanzania where the majority of the participants had moderate anemia 8.1% followed by mild 7.6% then severe anemia that was 2.3% of all participants (4). However, findings from this study were found to be the opposite of what was found in the studies done in Gondar, Northwest Ethiopia (27), rural Jordan (28), and China (29) where the majority had mild anemia followed by moderate anemia. This difference may be the result of geographical variation of factors across different areas and the eating patterns of the participants.

Anemia among pregnant women was found to be statistically significant with pregnancy in the third trimester. Studies show that there is an increase in blood volume during pregnancy time which may lead to a decrease in iron storage. As the number of trimesters increases, the demand for iron in the body also increases therefore, there is a great chance for those who are in the third trimester to develop anemia compared to those in the first trimester. These findings are supported by the studies done in a tertiary referral hospital, Northern Ghana, and (30). Pumwani Maternity Hospital, Kenya (31). The study also found that anemia was more prevalent in women who started their ANC visits in the second or third trimester. These women are likely to get iron and folic acid supplementation for a shorter duration during pregnancy as compared to those who started attending ANC earlier. This may have contributed to the high prevalence of anemia recorded in this study (31). The association was statistically significant.

Not consuming vegetables, meat, eggs, and fish were significantly associated with being anemic. This can be due to the fact that these are iron-rich foods and, hence, little or no consumption of these foods can be an important contributor to anemia. The studies done in the Volta Region, Ghana (32), and northern central Ethiopia (33) support these findings. Inadequate intake of micronutrients in food-insecure households can be a result of under-consumption of food or overconsumption of energy-dense but nutrient-poor diet which are becoming increasingly cheaper sources of calories for consumers who are poor (34). The current study also reported inadequate dietary diversity, which was significantly associated with anemia. Food diversity is advised to pregnant women since it is a period which demands physiologically higher nutrition than usual. Similar findings were reported from the study done in West Ethiopia (35) and from Southern Ethiopia (1). Other contributing factors to higher prevalence may be low income and the number of children. These factors are commonly cited in a number of studies (4, 8, 10, 14, 36).

### Strength

The study's findings are useful in strategizing an anemia prevention program within the Mkuranga district.

### Limitation

The study was institutional-based. To strengthen the findings, further community-level studies should be conducted. Also, the study excluded those who were severely ill and unable to respond due to difficulty in obtaining the venous blood sample. This may have reduced the prevalence of anemia.

Recalling bias since some of the participants were having difficulties in recalling the diversity of meals that they took in previous months.

## Conclusion

Anemia is prevalent in more than eight in every 10 pregnant women attending ANC in Mkuranga district, Tanzania. Factors associated with anemia in these women included the third trimester, and non-consumption of vegetables, meat, eggs, and fish. It is necessary to educate pregnant women about sources of iron and ways to improve its absorption while clarifying factors that contribute toward the risks of anemia and the importance to take iron supplements during pregnancy. Health care providers should inform pregnant women as well as women of reproductive age about sources of iron-rich foods. Health education on risk factors should be promoted to create awareness of the prevention of anemia.

## Data Collection Tools and Questionnaire

Data collection methods included a structured questionnaire and blood sampling.

### Blood Sampling

The machine that was used for lab work is called **CelltacEs Nihon Kohden, and** it was perfectly calibrated. To ensure the accuracy of the machine, laboratory technician carried out a control test every day before starting the actual sample testing.

The procedure of collecting the sample for full blood picture test was as follows:

1. Participants were required to go to the laboratory where they were instructed to sit upright on a chair and rest their arms face up on an elevated armrest. The laboratory technician applied a strap tourniquet around the top of their arms to temporarily restrict the blood flow from the arm back to the heart. This made the vein inside of the client's elbow dilate and, therefore, easier to access.
2. The area where the needle was inserted was wiped with a sterile alcohol wipe to reduce any risk of infection. A needle was inserted into the vein and a small amount of blood (4 cc) was drawn into the vial attached to the needle.
3. After the procedure, the laboratory technician applied with pressure a small cotton pad on the entry point to stop the flow of blood. The pad was strapped on with a band-aid. Finally, the participants were instructed that the pad was only needed to remain on for a couple of minutes.

The findings obtained were recorded in the participant questionnaires. Based on WHO guidelines, a Hb level <11 g/dl is indicative of anemia.

### Blood Sample Transportation

Blood samples from Irene-Kilimahewa Health Center were collected and kept in the cool box with the ice packs of 2–4°C and then transported to Mkuranga District Hospital where the test has been conducted. The samples were tested within 8 h after it was collected.

### Structured Questionnaire

A structured questionnaire was used for collecting data that was assessing factors associated with anemia in pregnancy. The questionnaire was developed from standard closed-ended questions that were adopted from TDHS-MIS 2016 for demographic and socio-demographic characteristics, and socio-economic characteristics, Household food insecurity access scale (HFIAS) to measure participants' household food access and household dietary diversity score (HDDS) to measure food diversity. Also, some other validated questions from the previously validated tools were adapted to measure disease burden. The questionnaire was written in English then translated to Kiswahili. All references are displayed in the reference list.

#### Pre-testing of the Questionnaire

The questionnaire was pretested at the ANC in Irene Kilimahewa health Center, Mkuranga. Pregnant women interviewed during pretesting found the instructions and the language of the tool to be clear and understandable, and that the time taken to complete the questionnaire was 30 min. Pregnant women involved in questionnaire pretesting were not allowed to participate in the study.

## Data Availability Statement

The raw data supporting the conclusions of this article will be made available by the authors, without undue reservation.

## Ethics Statement

The studies involving human participants were reviewed and approved by Muhimbili University of Health and Allied Science University Senate and District Executive Director at Mkuranga District. The patients/participants provided their written informed consent to participate in this study.

## Author Contributions

EN designed the study, conducted data collection, did data analysis and interpretation of findings, and wrote and approved the manuscript. SM and BS provided technical inputs to improve the design of the study, supported data analysis, read, improved, and approved the final manuscript write-up. All the authors read and approved the final manuscript.

## Funding

EN obtained the scholarship and fund to conduct a study from the Swedish International Development Cooperation Agency (Sida) in collaboration with Muhimbili University of Health and Allied Sciences (MUHAS).

## Conflict of Interest

The authors declare that the research was conducted in the absence of any commercial or financial relationships that could be construed as a potential conflict of interest.

## Publisher's Note

All claims expressed in this article are solely those of the authors and do not necessarily represent those of their affiliated organizations, or those of the publisher, the editors and the reviewers. Any product that may be evaluated in this article, or claim that may be made by its manufacturer, is not guaranteed or endorsed by the publisher.

## Abbreviations

ANC: Antenatal Care
AOR: Adjusted Odds Ratio
CI: Confidence Interval
Hb: Hemoglobin level
HDDS: Household Dietary Diversity Score
HFIAS: Household food insecurity access scale
IPTp: Intermittent preventive treatment in pregnancy
ITN: Insecticide-treated bed nets
OR: Odds Ratio
RCH: Reproductive and Child Health
TDHS-MIS: Tanzania Demographic and Health Survey-Malaria Indicator Survey
WHO: World Health Organization.
